# Surgical Memoirs of the Campaigns of Russia, Germany and France

**Published:** 1832

**Authors:** 


Art. IX. *Surgical Memoirs of the Campaigns of Russia, Germany, and France.* By Baron D. J. Larrey, Surgeon-in-Chief of the Hospital of the Royal Guard, Ex-Inspector General of the Military Medical Staff, Ex-Prime Surgeon of the Grand Army, in Russia, Saxony, &c. Translated from the French, by John C. Mercer, of Virginia, Student of Medicine. Philadelphia: Carey & Lea, Chesnut street—1832.

The name and services of Baron Larrey have been long and advantageously known to the Medical Profession through the translation of three volumes of his Surgical Campaigns, by Dr. Hall. Few individuals have had greater opportunities for acquiring a practical knowledge of surgery, or are better qualified to improve those opportunities for advancing the interests of science.

He entered the French army as assistant surgeon in 1787, and in consequence of his great merits he was in a few years advanced to the rank of surgeon-in-chief of the army. For nearly twenty years he was almost constantly with the principal division of the army, and was present at nearly every great battle.

Bonaparte, who delighted in the exhibition of that vigor and decision of character which elevated himself so much above the rest of mankind, took a peculiar pleasure in having Baron Larrey near himself in all his campaigns. It devolved upon him to superintend every thing connected with the military hospitals, and to direct the arrangements for affording relief to the wounded on the field of battle; and it is no disparagement to the Emperor to say, that the talents which Larrey displayed upon these occasions were not inferior to those of his master. Always actively engaged in the duties of his station, he manifested, in conjunction with the virtues which qualified him for the perilous and trying scenes in which he was called to act, the amiable virtues appropriate to his profession. In every engagement he took upon himself the performance of the most important operations. These frequently amounted to fifty or sixty, and upon one occasion to upwards of two hundred, and he was always in readiness, with an indifference to danger worthy a follower of Napoleon, to transport himself to any part of the field where his presence was called for.

His memoirs are written after the model given by Ambrose Pare, giving an account of every thing he met with in the course of the campaign: natural curiosities, geographical descriptions, improvements in the arts, are arranged together with military movements and surgical operations. Scarcely had the author improved the leisure which the short interval of peace, before Napoleon's unfortunate Russian campaign, afforded him to finish three former volumes of his memoirs, when he was suddenly called upon to renew his labors.

It is not consistent with the design of this work to follow the author through the detail of his campaigns. The desultory manner of the essay renders it difficult to give any thing like an analysis of the surgical adventures, and we shall therefore be compelled to confine ourselves to the account of a few of the important operations and scenes of thrilling interest with which those remarkable campaigns that immediately preceded the downfall of Napoleon abounded.

While the troops were yet in Poland preparing for more serious operations, the Baron had opportunity of observing some cases of *Plica Polonica* in individuals who had long been languishing in hospitals. In all these patients he discovered traces of inveterate scrofulous or venereal taint, and the head was covered with thick masses of hair, loaded with filth and vermin, suffered to remain for the purpose of producing a salutary crisis to the constitutional disease, and urged in vain upon the attendants to remove the hair, and subject the patients to an alterative treatment based upon the mercurial preparations. "This advice was rejected, and each party adhered to their respective opinion, thus exemplifying the very great difficulty with which prejudice is eradicated."

While the army was yet in Poland, some remarkable wounds were met with—one given in the last number of this Journal,* in the person of a Russian officer, in whom *the whole of the nose, the upper lip*, and the *palatine arch* were separated from the face, and left adherent by two small shreds of the upper lip at the commissures. The parts were replaced and reunited without deformity. A French officer received a musket ball in his bladder, from which it was extracted by the operation of lithotomy; the patient recovering in thirty days. A third wound no less remarkable occurred in a Russian soldier, whose forehead was pierced by an iron ball weighing seven ounces, which lodged in the anterior part of the brain. This patient also recovered. To these wounds allusion will be made hereafter. The following extract gives a striking picture of the destitution of the surgical department of the army after the bloody battle of Smolensk, when the wounds of nearly twenty thousand men were to be dressed without an article of the necessary materials, a destitution that was excelled only by the ingenuity and perseverence which supplied the defect.

"Our situation here was similar to that at Witepsk, in relation to the deficiency of all kinds of materials for dressing the wounded. It be-

* Vol. VI. No. 1, p. 156.

came necessary for me in this, as in many other instances, to discover substitutes for such means as were wanting. Thus, in lieu of linen, which we had exhausted during the preceding days, I employed the paper found in the building which we converted into an hospital, and which contained the archives of the city. Parchment served the purpose of splints and splint cloths, tow, and the down of the birch tree (*betula alba*) were used in the stead of charpee, and paper for covering the patients. But, what difficulties was it not necessary to surmount? what toils did we not endure at this conjuncture? Nearly all the inhabitants of the city had abandoned their houses, and the majority of those, which offered any resources, had been a prey to flames and pillage. I was actively aided in all these labors by the surgeons of the *ambulances* attached to head-quarters, and by those of the guard. We were occupied both during the night and day, in dressing those wounds that were caused by the sword and fire, and notwithstanding the scarcity of means in our possession, every necessary operation was performed in twenty-four hours."

The Baron, during this campaign, amputated the arm at the shoulder joint very frequently, and with great success. The following case is remarkable as an instance of the power of the constitution to resist the consequences of the most severe wounds.

"One of the most remarkable of these injuries, was that sustained by a corporal of the thirteenth regiment of the line. A bullet, of large size, shattered the head of the left humerus, the clavicle, and the whole of the scapula. The bony fragments were thrown posteriorly upon the back, and the soft parts abraded and lacerated. The wound presented a very unfavorable aspect. This soldier, in a state of unsupportable agony, loudly demanded the removal of the remaining portion of his arm, and the numerous osseous splinters, driven into its muscular parts. Notwithstanding the slight hopes inspired by the situation of this unfortunate individual, I attempted the following operation. After having removed the arm, which was attached to the body merely by some shreds of flesh, and tied the axillary artery, I extracted all the osseous fragments detached from the muscles and periosteum. The principal disorganized soft parts were then excised, and the lacerated and unequal edges of this extensive wound approximated, and maintained in this state of contiguity by means of adhesive straps and a large piece of linen, dipped in a solution of gum arabic, rendered stimulating by the chloride of sodium. The dressings were terminated by the application of fine tow, and the scapular bandage. The patient, after experiencing evident relief, was confided to the care of M. Sponville, one of the surgeons-major of the light *ambulances*. I was subsequently informed, that he was removed on the thirty-fifth day from Smolensk to Poland, in a state progressing to a cure. I have since had no tidings respecting him; but there is no doubt of this soldier's recovery, provided he escaped other maladies."

The celebrated battle of Mocowa, the last stand made by the Russians in defence of their capital, was fought on the 7th Sept., commencing at 6 o'clock, A. M., and continuing until 9, P. M. Baron Larrey, in consequence of the limited number of surgeons high in rank, was compelled to perform most of the important operations himself. A square space, about 1000 yards in circumference, was assigned to the medical staff, where they continued the performance of difficult operations in the open air without intermission, except when occasionally called to dress the wounds of a superior officer on the field of battle, until late in the night of the following day. This situation was the more distressing on account of the cold cloudy weather. "It was with difficulty that a wax taper was kept burning before me during the night. I had no absolute need of it," he adds with a very excusable vanity, "but for the application of ligatures to arteries."

In a number of cases upon this occasion he performed the operation of amputating the thigh on a level with the great trochanter, where the wounds were so high as to prevent the circular operation. One patient, whose thigh was amputated at the hip joint, recovered.

He insists strongly on the general propriety of amputating when the os femoris is broken by a gun-shot wound. The necessity is very urgent when the ball has pierced the limb a little above the knee. The wound at first view appears slight, and an inexperienced surgeon would think every thing favorable to the preservation of the limb, the patient for the first few hours suffering but little; but in a few hours tumefaction supervenes, the cellular substance becomes filled with black grumous blood, and the constitution sinks under gangrene or protracted suppuration.

The Baron preserved the knee in many patients in whom the leg was wounded by gun-shot wounds, so as to induce most of the surgeons of the army to recommend amputation of the thigh.

In these cases he sawed through the tibia, above the level of the tuberosity in which the patella is inserted, having previously dislocated the fibula. These cases terminated happily, although the integuments were not at all times sufficient to cover the wound. Several of the patients fabricated for themselves wooden legs, and made good their retreat through all the horrors of that ill-fated expedition. During the first twenty-four hours Baron L. performed about two hundred amputations. Many of these perished miserably from the want of every necessary which their unhappy condition demanded. Horse flesh, potatoes, and the stocks of cabbage, were for some days the only aliment, and even these were for a time exhausted.

These distressing privations were relieved upon the arrival of the army in Moscow; which city they found deserted and in flames. In the retreat, the wounded that were unable to take care of themselves all perished.

Two chapters of the work are devoted to an account of the retreat, and the history of the effects of cold upon the army. The army commenced their retreat on the 19th of October, and from this period until the middle of December, they suffered every distress that cold, hunger, and every species of privation can inflict upon men effecting a retreat in the depth of winter, through a hostile country, naturally barren, and still further desolated by the ravages of war. "Three thousand men, consisting of the best soldiers of the guard, as well infantry as cavalry, nearly all of whom were from the *southern part of France*, were all that resisted the cruel reverses of the retreat. They still preserved their arms, their horses, and their warlike appearance. The honor and renown of the French armies were in a measure concentrated in this small chosen body."

Baron Larrey states as a curious fact, that the troops from southern climates withstood the morbid effects of cold much better than those, who by a habitual exposure would be expected to be less affected by it.

"I have remarked, that individuals of a dark complexion, and of a bilio-sanguine temperament, almost all from the southern countries of

Europe, resisted the severe cold better than those of a fair complexion and lymphatic temperament, who, with few exceptions, were inhabitants of northern situations. This is contrary to the generally received opinion. The circulation in the former is doubtless more active, and the vital forces possessed of more energy. It is also probable that their blood preserves much better, even under the influence of the most intense cold, the principles of animal heat, which is identified with its coloring portion. From the same cause, their moral powers are sustained to a greater degree, courage does not abandon them, and by a careful preservation of themselves, they can shun dangers better than the inhabitants (who are in general phlegmatic) of cold and humid climates. Thus we saw the Hollanders of the third regiment of grenadiers, belonging to the guard, consisting of one thousand seven hundred and eighty-seven men, both officers and soldiers, almost totally perish, for there returned to France two years subsequent, but forty-one of the number, comprising the colonel, General Tindal, who was wounded. The other two regiments of grenadiers, however, consisting of men, who were, with few exceptions, from the southern provinces of France, preserved a pretty large part of their soldiers. It is, moreover, true, that in proportion to their numbers, the Germans lost more soldiers than the French. Several of our physicians that remained at Wilna have assured me that the cold destroyed more individuals of the coalition, proportionally speaking, than of the French, (as I have already remarked;) though the former had access to a greater variety of means of guarding themselves against the effects of this deadly agent, than our unfortunate countrymen. The latter, deprived of their garments by the Cossacks, and driven from place to place in a state of more or less complete nakedness, did not resist in a less degree, the greater part of the injurious effects, resulting from the cold air, and, by dint of courage and industry, obtained for themselves an immunity from total congelation.

The French, Portuguese, Spaniards, and Italians, were again those, from whose ranks was derived the smallest number of victims to these cruel reverses. This is a new argument against the assertion of the author of the *Esprit de Lois*, a new proof, that the inhabitants of these southern countries have more energy and power to resist the action of cold, than those of northern locations. Agreeably to the report of several physicians and surgeons, who participated in the fate of our soldiers, and were conveyed with them to Siberia, nearly all the individuals belonging to our allies from Germany, Hanover, and Holland, perished at an early hour. Some Russian troops and Poles, however, resisted these calamities much better. But, as I have remarked in my campaigns when speaking of it, this latter nation originated in Asia Minor, and, according to this report, must have great similitude of Physical constitution and character to the inhabitants of the southern countries of Europe, such as the French. Thus, whatever may be the governments and laws, under which the inhabitants of southern countries live, they will always be superior in activity, moral energy, and physical constitution, to those, whom cold and permanent humidity must, by restraining their energies, keep in a constant state of apathy, indolence, and a kind of timidity. Spain furnished us a signal proof of this truth.

Doctor Mestivier, who lived several years at Moscow, assured us, that the French alone could walk with impunity, in the streets of that city, during the most intense cold of winter, with a simple great coat over their other garments, whereas the inhabitants could scarcely withstand the severe weather, although they were covered with furred cloaks."

We transcribe the following account of the manner in which the death of these men took place.

"The death of these unfortumate victims was preceded by paleness of the countenance, by a kind of delirium, difficulty of speech, weakness of sight, and even entire loss of this sense. In this state some walked to a greater or less distance, supported by their comrades or their friends. Muscular power was sensibly diminished, and these persons staggered like men under the influence of intoxicating liquors. Their debility progressively augmented until they fell prostrate, which was a certain evidence of the total extinction of life.

The uninterrupted and rapid march of the soldiers in a body obliged those that were incapable of keeping up with the progress of the troops, to quit the centre of the column, in order to resort to the edges of the road, and proceed along its sides. Separated from this compact body and abandoned to themselves, they soon lost their equilibrium, and fell in the ditches, filled with snow, from which they arose with difficulty. A painful numbness soon seized upon them, and lethargic drowsiness supervening, their sad existence was soon terminated. There frequently occurred, previous to death, an involuntary discharge of urine. In some instances, hemorrhage took place from the nose, a circumstance that was particularly remarked by us on the heights of Miedneski, which appeared to me to be one of the most elevated points of Russia. I have grounds for believing, that the barometer would have fallen considerably in this high situation. The exterior atmosphere having become rarefied, and the elevation of this region offering no resistance to the action of the fluids, the movements of which are supported by the internal vital powers and the expansion of animal heat, they escape at those points where they meet with the least resistance. It is commonly from the mucous surfaces and particularly that of the nasal membrane, in which the capillaries are very abundant and susceptible of prompt dilatation, that these discharges take place.

This death did not appear to me to be attended with much suffering. the vital powers were gradually extinguished, and together with them the general sensibility was removed, and the perceptions of the sensitive faculties lost. It is probable, that the heart was at last paralyzed, and all the vital organs arrested at the same time in the performance of their functions. The fluids, already reduced in quantity by privations, and the absence of caloric, speedily coagulated. We found nearly all of those, who perished under the continued influence of cold, lying on the abdomen. Their bodies were stiff, their limbs inflexible, the skin discolored, and without any appearance of a gangrenous spot."

Immense numbers perished in consequence of imprudently approaching bivouac or suddenly entering a warm room. Some fell dead suddenly; in others gangrene manifested itself, and spread with such rapidity, that its progress was visible to the naked eye, and in a third class the limbs suddenly swelled, the turgescence appeared to pervade the pulmonary tissue, and the patient died of suffocation in a few hours.

The means recommended by Larrey for the prevention of mortification, were dry friction with snow, or substances possessing little heat, and if this failed in restoring the circulation, the immersion of the limb in cold water, in which it was directed to be bathed until bubbles of air were seen to disengage themselves from the congealed part. By the use of these means he escaped any serious accident, although his fingers and toes were frequently deprived of all sensibility.

No internal maladies manifested themselves among the soldiers during the retreat, but when they came within the confines of ancient Prussia, where they had access to food placed at their discretion, and were placed in warm rooms, the majority of them were seized with ataxic fever, accompanied with inflammation of the membranes, especially of the meninges and the mucous membrane of the air passages.

"These two principal effects were announced by compressive pains in the head, accompanied with heaviness, injury of the mental faculties, and alteration in those of the sensual organs. The patient was seized with general debility, and extremely painful anxiety. Cough manifested itself and rapidly augmented. It was more or less violent, and attended with a mucous, and occasionally a bloody expectoration. There frequently supervened, at the same time, diarrhœa, with an inclination to vomit, and colicky pains. The pulse was febrile, the skin dry, and the patient experienced a painful numbness in the limbs, cramps, convulsions, and a stinging pain in the soles of the feet. The patient slept with difficulty, and was subject to sinister dreams. The vessels of the conjunctiva were injected. Paroxysms of fever were manifested during the evening. The pulsations of the carotid, and temporal arteries, became sensible to the eye, and delirium, or lethargic drowsiness was established, and the danger of the sufferer became imminent.

In the inspections of the dead bodies which I frequently had an opportunity of making, a white layer of albuminous substance, unconnected with any suppurating point, was found on the surface of the brain. The sinuses of the dura mater were filled with black coagulated blood. The encephalon was compressed, its tissue more dense than in ordinary cases, and its vessels injected with dark colored blood. The mucous membrane of the larynx and bronchia was of a blackish brown tint in some parts of its surface, the intestines considerably shrunk, and the omenta scarcely observable; the latter was the result of abstinence. Gangrenous eschars were perceived in almost every instance, in the inferior extremities and abdomen."

A favorable termination was indicated by nasal hemorrhages, or by a dysentery supervening between the fifth and ninth day, and in some cases by profuse sweats staining the linen of a brownish hue. An unfavorable issue, on the other hand, was indicated by apoplexy, erysipelatous spots on the lower extremities which soon became gangrenous, the urine becoming scanty and of a dark color.

These cases were treated by cupping and leeching during the stage of excitement, and occasionally by drawing blood from the jugular vein or the temporal artery. Good effects also resulted from the circumspect application of ice to the head. Pediluvia and sinapisms were applied to the feet, and embrocations of camphorated vinegar over the whole surface of the body, at such a temperature as was indicated by the state of the skin. When the excitement was reduced, a gentle emetic was given with great advantage. After the primæ viæ were cleansed, the patient was placed on the use of mild tonics, and a nutritive diet.

The individuals who labored under this disease had a tedious convalescence, a consequence of the derangement and debility of function, and the long abstinence, produced by the excessive privations to which they had been exposed, and were under the necessity of returning to their accustomed habits with extreme caution. They were obliged to eat often, and a little at a time, as the least excess in eating gave rise to colics, and speedily occasioned relapses. The convalescence was followed in many cases by the loss of hair from every part of the body, and in one remarkable case by the falling off of the nails from the hands and feet. These were, however, after a time regenerated.

In the spring, the war was again renewed in Saxony, and this campaign forms the subject of the fifth chapter. We cannot indulge ourselves in giving an account of the important surgical cases which resulted from the numerous and bloody battles of this campaign.

He describes an absurd mode of amputating limbs, in practice with the Saxon, Prussian, and Polish surgeons. We give the description in his own words.

"The Saxons divided the skin and muscles by means of a curved knife at one stroke, and the bone nearly on a level with the section of the soft parts. The tourniquet exercising a strong compression on the arteries, the wound was sewed up without the application of ligatures to the vessels. The exact and close union of the edges of the wound generally prevented hemorrhage. Notwithstanding the simplicity and reasonableness of the French method, nothing less than experience and the success obtained, could convince the physicians of the country of its utility, who, in other respects, were very estimable for their social qualities and distinguished merit."

He was induced to inquire into the practice in consequence of observing the patients who had suffered amputation, labor under excrutiating pain. He advised the immediate removal of the ligatures and of the adhesive straps by which they were supported, and the application of emollients to the stump—nevertheless, many of the patients died of irritation or gangrene.

A curious question was proposed to Baron Larrey during this campaign, for his decision. The proportion of wounded in this campaign had been unusually great, and of these, a very considerable number consisted of mutilations of the fingers and hands. A rumor consequently gained circulation, which was countenanced by many of the surgeons, that the soldiers had voluntarily maimed themselves to avoid further service. The author supposed that the large proportion of this description was occasioned by the more elevated position of the enemy, who, in these engagements generally occupied the summits of the hills. The balls glancing from the barrels of their guns, would consequently be more liable to wound their hands, as the more exposed parts. A similar event had been observed upon other occasions, especially in the war which had been conducted in the mountainous districts of Spain.

This chapter contains a valuable memoir upon wounds of the head. A short quotation will exhibit the subjects to which he confines his remarks.

> "First, Those cases in which trephining is indispensable, and the period at which this operation should be performed;
>
> Secondly, Those instances in which the trephine, though as strongly recommended by authors, is useless, even injurious, and the means that may be adopted, under some circumstances as substitutes for this operation;
>
> Third, What is expedient in hernia of the brain;
>
> Fourth, and finally, the causes of absceses in the liver, succeeding wounds of the head."

Trephining is indispensable under the following circumstances.

> "In a wound of the head, accompanied by a fracture of the cranium, should the fragments of bone be displaced and driven internally, so as to injure the brain and dura mater, the trephine is indispensable.
>
> When the foreign body, which has caused the wound, is enclosed between the pieces of bone, or has penetrated into the interior of the cranium, without being removed from the vault of this cavity, the case is again one which demands the application of the trephine.
>
> Finally, when the surgeon is assured of the existence of effused fluid under the cranium, this instrument is also indicated."

We before alluded to the case of the Prussian soldier, whose frontal bone had been penetrated by an iron ball weighing seven ounces. The patient experienced a sensation of weight, and an extremely painful dullness in the head, and fell into a state of syncope when he inclined backward. He kept himself constantly in a sitting posture, resting his head upon his knees. The trephine was applied, the ball and some fragments of bone removed, and the patient did well. In wounds of this kind, if the subject be young, the bones yield, and permit the ball to pass through a very small perforation, consequently, the trephine is indispensable for its extraction.

2. The trephine is not to be applied when the fragments of bone are not driven in, when no foreign body is present, and when the symptoms of compression are not very urgent. When the trephine is necessary, it should be applied before the inflammatory symptoms supervene, and if this period is suffered to pass unimproved, it should be delayed until the inflammation subsides. If this event does not take place, the only effect of the operation would be to hasten the fatal issue.

3. Hernia of the brain (encephalocele). The Baron, in this disease, is opposed to the knife, caustic, pressure, or stimulating applications. He has seen but one case terminate in recovery; and in this, the hernia was not very large. The dressings, in this case, were of the mildest kind, the protruding brain was carefully protected from the air, and the causes of internal excitement, and external mechanical irritation carefully avoided.

The plan proposed by Professor Dudley, appears to hold out a reasonable prospect of success. He recommends the hernia to be covered with a piece of very soft dry sponge, which is bound on with a moderate degree of firmness. The sponge gradually expands by imbibing the secretions from the wound, and thus a moderate compression is kept up, which restrains the protrusion, and at length, by degrees, reduces it without violence.

We subjoin a curious instance of the gradual recovery of the intellectual powers after severe injury of the brain. A soldier was wounded with a small sword, which, entering the left canine region near the ala nasi, penetrated backwards, and inwards, three and a half inches, doubtless entering the cavity of the cranium through the ethmoid bone, and producing effusion there. The senses were destroyed, and the right side of the body paralysed. These have been gradually returning, although in an imperfect and unequal degree.

"The recollection of substantive nouns, which bore much analogy to proper names, was totally destroyed, and is not at present regained but with very great difficulty, whereas the memory of images, and every thing susceptible of description is in a state of perfect integrity. Thus, for instance, the patient recalled very readily the person and features of M. Larrey, whose services he had frequently received in different diseases and wounds. He knew him very well, and saw him constantly before his eyes, (expressions used by the patient,) but he could never recollect his name, so that he designated him by that of M. Chose. He also forgot the names of his relations and friends.

*Abscesses of the liver following wounds of the head.* Different hypotheses have been framed for the purpose of affording an explanation of this curious phenomenon. The most plausible has been, that the lesion of the liver is produced by the same accident that produced the injury of the head, and the results of experiments on dead bodies, have been given in defence of this hypothesis; but experiments on dead bodies can seldom explain the phenomena which occur during life. The size, weight, and delicate organization of the liver and the insufficient manner in which it is supported, have been supposed to render it peculiarly liable to injury from a violent shock given to the body, but this organ owes its security to the yielding character of its adhesions, and the vital resistance of the abdominal parietes, and hence it is generally found uninjured in post mortem examinations, after the most violent shocks, if the force of the blow has not been communicated to the hypochondrium.

The following is Baron Larrey's opinion, in support of which he advances a variety of evidence.

"1st, That these abscesses are but very rarely essentially owing to a blow, or direct pressure exercised on the liver, by the fall of the person injured, or by any contusing body, which may have acted violently on the right hypochondrium;

2d. That the origin of these abscesses should be referred to the sympathetic irritation, experienced by the viscus in which they are seated, in consequence of the inflammation established in the fibrous membranes of the cranium, or bones of the superior or inferior extremities, particularly those of the same side, and the determination towards this viscus of the ichorus miasm, or a fluid more or less acrid and subtile."

An account is given of a wound inflicted with the intention of committing suicide by discharging a pistol barrel charged with two balls, within the cavity of the mouth, which was cured without occasioning much deformity. It will be given under the head of *Analectic Intelligence.*

Some interesting wounds of the throat are detailed, one of which we extract on account of the practical principles involved.

"Jacques Brisnot, the second individual, was a junior sharp-shooter of the guard. He was wounded the day before the battle of the 27th of August, and was one of a small number of patients of this character, under the charge of M. Emangard, one of our surgeons-major. The danger of his situation was indicated by the very great embarrassment with which he breathed; his speech was nearly extinct, and he suffered the agonies of death. Respiration was scarcely carried on, and some bubbles of air, with a large quantity of frothy blood, escaped with difficulty, through a gunshot wound in the left side of the larynx, between the thyroid cartilage and os hyoides, which latter was fractured. The ball traversed the throat, and made its exit behind the left angle of the jaw. The patient lost much blood, and was nearly suffocated, in consequence of that contained in the cavity of the larynx; the epiglottis cartilage had been removed, and the thyroid cut. The attending surgeon-major hesitated as to the manner in which he should proceed to promote a restoration of the functions of respiration, and to cause the blood effused into the larynx, to issue from it. The very happy idea, however, of cutting the thyroid cartilage, occurred to him. At that moment, the air rushed with violence from the bronchia, and pushed before it the clots of blood, which filled this canal, and were about to cause the suffocation of the patient. The surrounding parts were relieved from their congested state, respiration re-established, and the life of the patient no longer in danger. The wound gradually assumed a healthy condition, the functions were successively performed with more regularity, the edges of the wound approximated, the cicatrix pretty speedily completed, and the patient cured in a very short time; merely a slight degree of aphonia remained.

This case proves the necessity of giving free vent to matters retained in the cavity of the larynx. In order to fulfil well the indicacation presented in contused wounds of this organ, with penetration of it, and extravasation of fluids, or the presence of foreign bodies, it is better to enlarge the wound on the side of the cavity towards the trachea, like the celebrated Spanish surgeon Virgilii, so far as the principal surrounding vessels or nerves permit, than to make an opening at one of the points selected for the performance of laryngotomy and bronchotomy. There would, in such a case, be two apertures in the larynx, instead of one, and the functions of respiration be thus injured.

The passage of balls into the muscular and lateral parts of the neck, however superficial may be the track formed by them, has almost invariably given rise to paralysis of the arm of the same side. Experience has taught me, that the laying open of the wound, executed with care, immediately after the accident, prevents this paralytic affection, doubtless because the nervous branches, lacerated or ruptured by the projectile, do not, as in the contrary case, contract too close adhesions at the depressed portion of the cicatrix, whence results an alteration in the vital properties of the injured nerves, and those with which they are connected. In consequence, also, of the binding force and compression exercised upon them, their sensibility is destroyed and paralysis is developed and propagated to all the parts to which the affected nerves are disturbed. Moxa, however, applied to the cicatrices, and along the course of the primitive branches of these nerves, restore their functions, and dissipate their paralysis. The employment of this remedy, in the treatment of this disease, has proved successful in many instances. The mode of using it is pointed out in another article of my *Campaigns*, and in the *Dictionaire des Sciences Medicales* under the head of *Moxa*."

*Wounds of the Thorax.* When a ball or other foreign body is lodged in the thorax, its constant tendency is to form for itself a passage downwards until it meets with a fixed point of support, whose sensibility is not greatly exalted by the extraneous irritation. In this manner it generally finds its way to the most depending portion of the chest, until it is supported by the diaphragm and the thoracic parietes. A fistula with a secreting surface is thus formed through the whole course of the foreign body, and a seat of purulent secretion is kept up also where it is lodged, giving rise to empyema, or troublesome fistulæ.

When the position of the projectile can be accurately ascertained, its immediate removal should be attempted. If the affection is recent, this may generally be done through the intercostal space, but when the case has been of longer duration these spaces become contracted, and the removal of a portion of one of the ribs becomes necessary. The form of the rib, and the nature of its connections render the saw or the trephine inapplicable. But the rib may generally be divided, without difficulty, by means of the lenticular saw used in trephining. The foreign body being removed, the suppurating cavity is soon closed. "The cavity is gradually obliterated by the enlargement of the capillary vessels of the pleura, mediastinum, diaphragm, and, perhaps, also, of a part of the lung: the intercostal muscles lose their contractile power, and the ribs approach each other; the cartilages are deprived of their curvature and depressed towards the thoracic cavity; the nutritive process in the sternum and ribs undergoes such a change, that these bony arches, having lost their curvature, assume a cylindrical form, and thus contribute to the reduction of the vacancy." In fine, every part in the vicinity contributes its share to bring the surfaces in contact, when their vessels contract adhesions, and form anastomoses; the cicatrization extends from the interior to the exterior, and the external wounds disappear.

*Wounds of the Abdomen.* We have little to notice under this head. The author recommends the dilation of the wound and the application of dry cups over it, and, if there be effusion of blood in the loose cellular texture which abounds in these parts, the application of cups with scarificatious in the vicinity. The cups, he thinks, have the power to remove the blood effused into the sinews of the wound, to relieve the debilitated vessels of their engorgement, to promote the absorption of the extravasated fluids, and to prevent inflammation.

In wounds of the abdomen, accompanied by protrusion of the omentum, if this membrane cannot be restored before the tumefaction, which quickly supervenes, takes place, the author recommends its restoration to the abdominal cavity to be left to nature. If it be strangulated, the external wound is to be laid open to a sufficient extent to liberate it completely, and the protruding part is to be carefully covered with fine linen, spread with a mild cerate, for the purpose of preventing its adhesion to the integuments around the wound, and of guarding it from the air and from foreign irritation. The tumor, in a few days, becomes dense, red, wrinkled, and highly sensible. These symptoms increase for three days, when they remain stationary until the fifteenth. At this period the sensibility and redness subside, the tumor diminishes in size, the angles of the wound are drawn apart, and it generally soon returns into the cavity of the abdomen. The rapidity of this reduction depends very much upon the age and constitution of the patient, and the situation of the wound. It takes place more readily in young and healthy patients and in wounds below the umbilical line. If it should be tardy, it may be accelerated by gradual and moderate pressure. If gangrene of the protruding omentum takes place, its separation is to be left to nature, simply removing such parts as may be taken away without touching the living parts. Temporary adhesive inflammation takes place between the omentum and edges of the wound. The affected part sloughs, and the pedicle, in a healthy condition, is quickly and spontaneously returned into the abdomen. Where the gangrene has invaded the viscera within the abdominal cavity, the patient must of course be left to the resources of nature, with the use of such means as may assist her operations.

These are the general principles upon which gangrene is to be managed. If a different course is pursued and the omentum is cut off to prevent the extension of gangrene, the life of the patient is endangered by hemorrhage, and, if a ligature be applied around a sound part of the omentum, severe irritation is produced, which is frequently followed by inflammation, abscesses, gangrene, and death.

The memoir on the wounds of the bladder contains much valuable historical information, in addition to the results of Larrey's experience. The bladder, when empty, is not easily wounded, either by a ball or cutting instruments. If it is penetrated above the peritoneum, the inflammation produced by the escape of the urine soon causes death. If the wound is below the peritoneal cavity, an emission of urine takes place from the wound, which will be continued or not, according to the position of the wound, and the care that is taken to keep the bladder empty. If the track of the wound is extensive, abscesses are formed in different parts, through which the urine escapes. In gun-shot wounds, however, this infiltration is not liable to take place until the separation of the sloughs.

Internal hemorrhages frequently takes place, and if they do not prove fatal by the loss of blood, serious consequences are often produced by its extravasation in the loose cellular membrane of those parts. The blood rarely coagulates in the bladder on account of its being mixed with the urine. In all wounds of this kind the author attaches great importance to the cautious dilation of the wound at the point of the entrance and exit of the ball. It prevents engorgement and inflammation, and facilitates the separation of the sloughs.

What is to be done when the projectile is lodged in the bladder? If the body is small, it may be passed by the urethra, and this event will be facilitated by dilating that passage. If they are larger, Baron Larrey recommends the operation of lithotomy to be performed at once. By this means we at once relieve the patient of the ball, and of other substances that may have been carried with it that would be likely to prove a nucleus for urinary deposits.

This volume contains several valuable memoirs—*on Wounds of the Arteries—on the Actual Cautery*, and *on Amputation of the Arm.* From these we shall only extract the Baron's mode of amputating the arm at the shoulder-joint.

"The patient being seated at a suitable height, I commence the operation by a longitudinal incision, beginning at the edge of the acromion and descending about an inch below the level of the neck of the humerus. By this act I cut through the integuments and divide the fibres of the deltoid muscle into two equal parts. Causing then the skin of the arm to be drawn towards the shoulder by an assistant, I form two flaps, an anterior and posterior one, by two oblique cuts, from within outwards and downwards, so that the tendons of the pectoralis major and latissimus dorsi are comprised in each section. No apprehension is to be entertained as to injuring the axillary vessels, since they are out of the reach of the point of the instrument. The cellular adhesions of the two flaps are then divided and raised up by an assistant, who at the same time stops the cut circumflex arteries, and the whole shoulder-joint is laid bare. By a third circular incision around the neck of the humerus, the capsule and articular tendons are divided; the head of the bone is drawn a little outwards, and the knife is passed to its posterior part in order to complete the section of the tendinous and ligamentous attachments on this side. The assistant immediately directs the first fingers of his two hands to the brachial plexus, in order to compress the artery and render himself master of the blood. The cutting edge of the knife is, in fine, turned backwards, and the bundle of the axillary vessels divided on a level with the inferior angles of the two flaps, and in front of the assistant's fingers. The patient does not lose a drop of blood, and, the pressure being continued, the extremity of the axillary artery is easily discovered; it is then seized with a pair of dissecting forceps, in order that a ligature may be immediately thrown around it. The circumflex vessels merely remain to be tied, and the operation is terminated.

The wound having been cleansed, the flaps are approximated, and maintained in contact by means of two or three adhesive straps, moderately tight, and the whole stump covered with fine linen dipped in some tonic liquid, such as warm wine. The dressing is concluded by the application of charpee or fine tow over the linen, with simple quadrilateral compresses, and an appropriate bandage."

At Troyes, in Champagne, I had occasion to operate on a soldier of the train of artillery. The case relating to this amputation has been drawn up and presented to the Society of the School of Medicine by M. Carteron, a physician of that city and witness of the operation.

In extirpating this soldier's arm, which a ball of large size had entirely disorganized, at the same time shattering the scapula, I extracted two-thirds and a half of the latter bone, and the humeral extremity of the clavicle. The details of the case may be seen in the Bulletin of this Academy.

Another instance, entirely similar, was presented at the capture of Smolensk in Russia; I performed the same operation and with like success. From the report which was addressed to me by M. Bachelet, surgeon-major of the hospitals of this place, I learned that the patient had been removed towards Poland, the wound having completely cicatrized.

How is the success obtained by us from this extirpation, according to the method which we have just described, to be explained? Such success, indeed, that of an hundred, and some operations of this kind performed by us in the different armies or in Paris, more than eighty, have terminated happily. This could be easily proved by the registers of the pension office with the minister at war. I will attempt to indicate the causes of it without, however, pretending to pronounce positively upon the question.

1st. The perpendicular incision which I make in the centre of the shoulder, marks out with accuracy the rest of the operation, and facilitates its execution; it is, moreover, done in a moment. The two flaps of the desired dimensions, are then cut in an exact and regular manner, so that this section presents the result of a circular amputation. The head of the humerus is easily dislocated in all possible cases.

By the manner in which I cause the vessels to be seized, previously to dividing the parts in the hollow of the axilla, I prevent all hemorrhage and place the life of the patient in perfect security; an appreciable advantage, on which essentially depends the success of the operation.

2d. Nature effects the cicatrization of flaps with so much the greater facility, as their union takes place in the direction of the longitudinal diameter of the glenoid cavity; the majority also of the muscles of the shoulder are divided lengthwise with their fibres, or cut at their tendinous insertions, a circumstance promotive of reunion, and of the formation of the cicatrix, which becomes linear.

3d. In short, we think that the few nerves belonging to organic life in the shoulder-joint, and the large number of them about the hip-joint, may in general explain the success of one of these operations, and in many cases the failure of the other."

The works of Larrey are among those which ought to be in the hands of every student of surgery. Independent of the advantage to be derived from tracing the mental operations and witnessing the resources of one of the most original observers of this or any other age, it is of the first importance to the student to imbibe the inspiration of genius by accompanying it through the emergencies which it is called upon to meet in a life of singular peril and self devotion.

Baron Larrey, as a surgeon, was exemplary for his humanity, which manifested itself in his attention to the soldiers, and the great exertions which he made, and the privations he voluntarily incurred for the sake of providing for their wants.

The attachment of the soldiers was consequently unbounded, and only equalled by the enthusiasm of their devotion to the Emperor.

The proof of esteem manifested in the following quotation, exhibited too at a time when almost all social feelings were annihilated in the instinctive impulse of self-preservation, confers upon the subject more honor than he has derived from all his professional achievements. The occurrence took place during the retreat from Russia, in the passage of the Berezina.

"The two bridges, the construction of which had been undertaken, were, in the mean time, completed without the knowledge of the enemy. The guard succeeded the first and fourth corps in their passage across them, and reached the opposite bank, without opposition and without accident. But as the cannons of large calibre were passing to the opposite side of the river, one of these bridges was broken down. The progress of the remainder of the artillery, of all the equipage, and *ambulances*, was thus arrested. The occurrence gave rise to great alarm among those that remained on the left bank. At this moment of suspended operations, the troops of Wittgenstein, which followed us closely, attacked our rear guard. All possible resistance was made by the latter. Being forced from its position, this body endeavored to effect their retreat, and facilitated by this movement the approach of the enemy, whose bullets and other projectiles fell among the immense crowd already collected at the head of the bridges. The latter had doubtless been repaired, but had become impassable from the impediments and disorder, of which they were the scene. Fear pervaded the minds of all. The multitude crowded, dashed on all sides, and rushed one against the other. The strong overpowered the weak, and the latter were trodden under the feet of the crowd. The carriages, artillery, and baggage wagons were overturned and broken to pieces, and the horses and drivers crushed under the wreck. In short, nothing was heard on all sides but lamentable shrieks. To complete this tragic affair, the badly secured bridges were again broken. From this moment, all hope of safety was apparently destroyed. The majority of the multitude were influenced only by despair. They rushed on a body of ice, supposing it possible to cross the river in this way, as the stream appeared to be congealed, but their progress was arrested, near the opposite bank, at a point, where the body of ice was interrupted by the force of the current. Some swam across this space, others had the misfortune to be drowned, or to become entangled against the sheets of the frozen element. They perished in this situation the more speedily in consequence of their being already benumbed by the cold, and attenuated by privations. Those possessed of more courage and prudence returned, in order to throw themselves into the power of the Russians, and withdraw from the horrid spectacle they were witnessing.

Many individuals, of all classes, lost their lives in crossing the Berezina. Mothers were seen voluntarily following the fate of their children, who had fallen into the river, or drowning themselves with their offspring, holding them tightly in their arms. Many other acts of a character equally touching, were observed during this calamatous state of things.

Notwithstanding the difficulties which were almost insurmountable, I had repassed one of the bridges some hours previously to its rupture, for the purpose of having some cases of surgical instruments, which were much needed by the wounded, conveyed to the right bank of the river. This short journey was near costing me my life. I had like to have perished in the crowd in my turn, when fortunately I was recognized. Every one immediately made exertions to aid my efforts, and carried from soldier to soldier, I found myself, to my great surprise, in a few moments on the bridge. This evidence of their attachment to me soon obliterated from my memory both the dangers, to which I had been exposed, and the loss of my baggage."

It seldom falls to the lot of any individual to receive so striking a testimonial to his personal worth. The gratification that he derived from it can only be conceived by him who is capable of making the sacrifices by which he merited so rich a reward. F.

